# Trimethoprim-Sulfamethoxazole-Induced Hyperkalemia in a Patient with Normal Renal Function

**DOI:** 10.1155/2012/815907

**Published:** 2012-12-13

**Authors:** L. Connor Nickels, Christine Jones, Latha Ganti Stead

**Affiliations:** ^1^Department of Emergency Medicine, College of Medicine, University of Florida, 1329 SW 16th Street, P.O. Box 100186, Gainesville, FL 32610-0186, USA; ^2^Division of Clinical Research, Department of Emergency Medicine & Neurological Surgery and Toral Family Foundation, Center for Brain Injury Research and Education, College of Medicine, University of Florida, 1329 SW 16th Street, P.O. Box 100186, Gainesville, FL 32610-0186, USA

## Abstract

The authors present a case of Trimethoprim-sulfamethoxazole-induced hyperkalemia in a patient with normal renal function. While toxicity of this drug has been reported in patients with renal insufficiency, this case highlights the toxicity associated with normal kidney function. Due to its popularity in the medical field and to the largely unrecognized effect of hyperkalemia, it is important to consider such adverse effects when prescribing TMX-SMX. One must be reminded of the possibility of the development of life-threatening hyperkalemia in relatively healthy patients.

## 1. Introduction

Trimethoprim-sulfamethoxazole (TMP-SMX) is a broad-spectrum antibiotic used to treat a variety of infections. Due to its efficacy, its ease of dosing, and to the relatively low expense, it has become a popular choice among many physicians. However, as with any medications, adverse reactions may be experienced. Rash and gastrointestinal upset are the most commonly reported side effects associated with TMP-SMX. In contrast, hyperkalemia is rarely reported and most physicians are not aware of this potentially harmful side effect. The literature has long reported the occurrence of TMP-SMX-induced hyperkalemia in patients with acquired immunodeficiency syndrome (AIDS), patients with end stage renal disease (ESRD), and patients on high dose TMP-SMX [1–11]. More recently, there have been reports of similar symptoms occurring in patients treated with standard dose TMP-SMX [12, 13], and in conjunction with other medications, such as enalapril and spironolactone [14–19]. We present a case of life-threatening TMP-SMX-induced hyperkalemia in a female with a normal creatinine whose only other identifiable risk factor was daily lisinopril.

## 2. Case

A 61-year-old female presented to the Emergency Department (ED) with a complaint of “I feel like I'm going to die.” She reported being in her usual state of health until seven days prior when she developed cold symptoms. She was prescribed TMP-SMX for her upper respiratory tract infection and had completed four days of the antibiotic course at the time of her arrival in the ED.

Upon examination, the patient reported two days of progressively worsening weakness and fatigue and one day of chest pressure and shortness of breath. Prior to arrival, she experienced an acute increase in the generalized weakness, rendering her unable to ambulate without assistance. Also, she reported nausea and diaphoresis. She denied any additional accompanying symptoms.

The patient's past medical history was significant for diabetes, hypertension, lupus, and hypothyroidism. Her current medications were metformin, lisinopril, methotrexate, and levothyroxine. Her surgical and social histories were unremarkable and she was not aware of similar illnesses in her family.

On physical exam, the patient was noted to be in extremis. Vital signs revealed a blood pressure of 190/65, a pulse of 100, and respiratory rate greater than 20. She was pale and diaphoretic in appearance. She was unable to sit upright on the stretcher without assistance or lift her extremities. Also, she was in moderate respiratory distress with tachypnea and increased work of breathing. Her breath sounds were coarse bilaterally. Cardiac exam was unremarkable for any pertinent findings other than tachycardia. On neurological exam, there was no focal deficit; however, there was significant generalized weakness throughout, 2/5 strength in all extremities. She was mentating normally.

Due to the patient's appearance, point of care (POC) testing was performed at the bedside. The results revealed a sodium level of 124, a potassium level of 8.3, a creatinine level of 1.0, a blood glucose of 361, and a troponin I level <0.10. The hemoglobin and hematocrit, as well as the venous blood gas, were all within the normal ranges. The patient's 12-lead electrocardiogram (EKG) showed a wide complex tachycardia with peaked T waves indicating hyperkalemic changes.

The hyperkalemia was treated immediately. The patient received calcium gluconate 1 gram IV, sodium bicarbonate 1 ampule IV, insulin 10 units IV, albuterol 2.5 mg/3 mL nebulized, and Kayexalate suspension 30 g/120 mL PO. She was started on a continuous infusion of insulin for her hyperglycemia. Shortly after, she experienced marked improvement in symptoms. Her chest pressure, nausea, shortness of breath, and diaphoresis resolved. The patient was able to move all extremities and was noted to have 4/5 strength in all four. Her EKG began to normalize. Nephrology was contacted for emergent dialysis.

Approximately two hours later, the patient experienced a return of all her symptoms. Labs were rechecked, showing the same findings of hyperkalemia with a normal creatinine. The hyperkalemia protocol was repeated. A vascular catheter was placed in the patient's right femoral vein for emergent dialysis access. The patient was admitted for further care.

After arriving in the ICU, dialysis was performed, and her potassium level decreased to approximately 6. Overnight, the insulin infusion was continued. The next morning, the patient received another dose of Kayexalate. Throughout the day her potassium level trended downward and normalized between 4 and 5, and her EKG changes resolved. During her stay, the patient's creatinine remained stable, and her renal ultrasound revealed no abnormalities of the kidneys. She also had an echo performed that showed an ejection fraction of 60–65%, and no wall motion abnormalities. It was determined that the patient's condition was due to TMP-SMX-induced hyperkalemia in the setting of daily lisinopril.

## 3. Discussion

Hyperkalemia is a dangerous condition, potentially leading to cardiac dysrhythmias and death. It is most often linked to renal dysfunction, but may be seen with adrenal disease, with diseases which cause destruction of muscle tissue, or with medications. Some of the most widely used medications have been implicated, such as potassium supplements, potassium-sparing diuretics, angiotensin-converting enzyme inhibitors (ACE-I), angiotensin II receptor antagonists (ARB), trimethoprim-sulfamethoxazole, and heparin [14, 20]. We focus on one drug in particular, TMP-SMX.

Some of the earliest cases of trimethoprim-induced hyperkalemia were reported in patients with HIV/AIDS. These patients are typically treated with large doses of TMX-SMX for Pneumocystis carinii [1–11]. It has been postulated that perhaps the declining renal function associated with the natural progression of the disease, combined with “high-dose” TMX-SMX is responsible for the untoward effect, as renal dysfunction accelerates the incidence of hyperkalemia [21]. However, more recent clinical studies have shown that hyperkalemia can be seen even with “standard-dose” TMX-SMX [15, 21–24].

One study in particular evaluated this relationship in immunocompetent patients in the outpatient setting [19]. This study looked at two groups: a treatment group and a control group. The treatment group was comprised of patients receiving “standard-dose” TMX-SMX which was defined as trimethoprim, 320 mg/day, and sulfamethoxazole, 1,600 mg/day. The control group included patients taking other antibiotics. The authors noted that the treatment group showed a statistically significant (*P* < 0.001) rise in potassium levels after 5 days of treatment. However, they reported only 6% of patients (3 patients) developed “severe” hyperkalemia, defined as ≥5.5, and 0 patients developed clinically significant hyperkalemia [19]. This led to the conclusion that life-threatening hyperkalemia occurs much less frequently in outpatients treated with “standard-dose” TMX-SMX than in patients with AIDS or renal insufficiency, who may be prescribed a “high-dose” therapy [19].

However infrequent the occurrence, it is important to understand the mechanism by which the adverse effect occurs. Trimethoprim is structurally similar to the potassium-sparing diuretic amiloride [20, 24]. It competitively inhibits the sodium channels of the epithelium in the distal nephron, thereby impairing renal potassium excretion [24]. This alone may induce hyperkalemia, but one must consider that the causes of hyperkalemia may be additive [14]. For example, studies have shown that TMX-SMX in combination with ACE-Is significantly increases the risk of hospitalization secondary to hyperkalemia [18]. This is likely due to the suppression of angiotensin II, which leads to a decrease in aldosterone levels. Since aldosterone is responsible for increasing the excretion of potassium, ACE inhibitors ultimately cause retention of potassium.

Due to its popularity in the medical field and to the largely unrecognized effect of hyperkalemia, it is important to consider such adverse effects when prescribing TMX-SMX. One must be reminded of the possibility of the development of life-threatening hyperkalemia in relatively healthy patients.
